# Black carbon particles in human breast milk: assessing infant’s exposure

**DOI:** 10.3389/fpubh.2023.1333969

**Published:** 2024-01-17

**Authors:** Charlotte Cosemans, Eva Bongaerts, Kenneth Vanbrabant, Brigitte Reimann, Ana Inês Silva, Eline Tommelein, Giulia Poma, Marcel Ameloot, Tim S. Nawrot, Michelle Plusquin

**Affiliations:** ^1^Centre for Environmental Sciences, Hasselt University, Diepenbeek, Belgium; ^2^Department of Pharmaceutical and Pharmacological Sciences, Experimental Pharmacology, Vrije Universiteit Brussel, Jette, Belgium; ^3^Toxicological Centre, University of Antwerp, Wilrijk, Belgium; ^4^Biomedical Research Institute (BIOMED), Hasselt University, Diepenbeek, Belgium; ^5^School of Public Health, Occupational and Environmental Medicine, Leuven University, Leuven, Belgium

**Keywords:** black carbon, human breast milk, public health, infants, air pollution

## Abstract

**Background/Aim:**

Human breast milk is the recommended source of nutrition for infants due to its complex composition and numerous benefits, including a decline in infection rates in childhood and a lower risk of obesity. Hence, it is crucial that environmental pollutants in human breast milk are minimized. Exposure to black carbon (BC) particles has adverse effects on health; therefore, this pilot study investigates the presence of these particles in human breast milk.

**Methods:**

BC particles from ambient exposure were measured in eight human breast milk samples using a white light generation under femtosecond illumination. The carbonaceous nature of the particles was confirmed with BC fingerprinting. Ambient air pollution exposures (PM_2.5_, PM_10_, and NO_2_) were estimated using a spatial interpolation model based on the maternal residential address. Spearman rank correlation coefficients were obtained to assess the association between human breast milk’s BC load and ambient air pollution exposure.

**Results:**

BC particles were found in all human breast milk samples. BC loads in human breast milk were strongly and positively correlated with recent (i.e., 1 week) maternal residential NO_2_ (*r* = 0.79; *p* = 0.02) exposure and medium-term (i.e., 1 month) PM_2.5_ (*r* = 0.83; *p* = 0.02) and PM_10_ (*r* = 0.93; *p* = 0.002) exposure.

**Conclusion:**

For the first time, we showed the presence of BC particles in human breast milk and found a robust association with ambient air pollution concentrations. Our findings present a pioneering insight into a novel pathway through which combustion-derived air pollution particles can permeate the delicate system of infants.

## Introduction

Human breast milk is a complex mixture of nutrients and bioactive compounds. It is the golden standard for infant feeding and nutrition and optimal for developing infants. The World Health Organization (WHO) and the United Nations Children’s Fund (UNICEF) recommend exclusive breastfeeding for the first 6 months of life for optimal growth, development, and health of the child (1, 2). Multiple studies reported various benefits of breastfeeding for children, including a decline in infection rates in childhood (3), lower risk of obesity (4), lower risk of type 2 diabetes mellitus (5), and lower risk of asthma (6). In addition, on a molecular level, being breastfed during infancy was associated with blood mtDNA content in adolescence. More precisely, the longer the adolescent was postnatally breastfed, the higher the blood mtDNA content was during adolescence, suggesting a duration-dependent effect of breastfeeding (7).

Unfortunately, concerns have emerged regarding the presence of environmental pollutants in human breast milk, and an increasing number of studies have reported their presence in human breast milk (8–12). A recent review by Martin-Carrasco et al. (13) described the presence of environmental chemicals, toxic metals, pesticides, mycotoxins, and per- and polyfluoroalkyl substances (PFAS) in both human breast milk and infant formula. Most of the published studies focused on the presence of environmental chemicals rather than particles, such as black carbon (BC). Environmental BC arises from the incomplete combustion of fossil fuels, wood, and biomass. BC is suspected to be one of the most harmful components responsible for the observed health effects from exposure to traffic-related air pollution (14). Not only were combustion-derived particles found in the frontal cortex of autopsy brain samples (15), kidney biopsies (16), and urine of healthy children (17), BC particles were also detected in placental tissue (18, 19), cord blood, and fetal tissues (i.e., liver, lung, and brain) (19). These findings show that BC has the potential to reach various organ systems. It is unknown whether infants are also exposed to BC via breastfeeding. Several rodent studies have already shown that other nanoparticles could be transferred to the offspring through breast milk (20–22). In fact, silver nanoparticles were distributed to breast milk and subsequently to the brains of breastfed pups (20). However, human studies investigating the presence of BC particles in human breast milk are lacking. Therefore, this study aims to explore the presence of these particles in human breast milk and assess preliminary associations with air pollutants. By shedding light on this issue, we can contribute to the ongoing efforts to mitigate exposure, promote a healthier environment, and ensure future generations’ optimal growth and development.

## Materials and methods

### Experimental protocol for BC detection in human breast milk

Eight breast milk samples expressed with a breast pump were obtained from non-smoking lactating mothers who voluntarily provided the samples in polyethylene breast milk bags. Samples were sealed and stored at −20°C until further analyses. Ethical approval (B371201216090) for this study was obtained from the Ethical Committees of Hasselt University (Diepenbeek, Belgium) and the East-Limburg Hospital (Genk, Belgium), with all participants providing informed consent. The study protocol was conducted in compliance with the Declaration of Helsinki.

BC particles from ambient exposure present in breast milk were detected using a specific and sensitive detection technique based on the non-incandescence-related white light generation of the particles under femtosecond illumination, as previously described (23). To minimize contamination of the samples, pre-analytical quality control included washing steps of the imaging chambers and blank measurements of these chambers, resulting in no detectable BC particles. Every step was designed and monitored to preclude any possible contamination. We previously confirmed the carbonaceous nature and tissue embedment of the identified BC particles using rigorous validation experiments (18, 19) and performed BC fingerprinting in human breast milk using the reference particles (i.e., conductive carbon black (CCB); 2 μg/mL in Milli-Q; US Research Nanomaterials, Houston, TX, United States). Images of the human breast milk were collected at room temperature using an inverted Zeiss LSM880 confocal microscope (Carl Zeiss, Oberkochen, Germany) equipped with a femtosecond pulsed laser (810 nm, 120 fs, 80 MHz, MaiTai DeepSee, Spectra-Physics, Santa Clara, CA, United States) tuned to a central wavelength of 810 nm using a Plan-Apochromat 20x/0.8 (Carl Zeiss). Two photon-induced white light emission by carbonaceous particles was acquired in the non-descanned mode after spectral separation and emission filtering using 405/10 nm and 550/200 nm band-pass filters. Each breast milk sample was shaken for half an hour and aliquoted at 50 μL per imaging chamber, and five-by-five tile scans were collected 1 μm inwards from the bottom of the imaging chamber (i.e., 170 μm thick 24 × 24 mm coverslip). The resulting tile scans had a field of view of 2124.8 × 2124.8 μm^2^ containing 25 images with a 2,560 × 2,560 pixel resolution and were recorded with a 1.54 μs pixel dwell time at nine different locations in the imaging chamber. All images were acquired by ZEN Black 2.0 software (Carl Zeiss). An automated and customized MATLAB program (MATLAB 2010, Mathworks, Natick, MA, United States) was used to count the number of black carbon particles in the tile scans recorded for each breast milk-filled imaging chamber (18). First, a peak-finding algorithm detects connected pixels above a specific threshold value. Threshold values of 55 and 99.5% from the highest pixel intensity of the narrow 405/10 nm and broad 550/200 nm channels were used, respectively. These thresholds resulted in reproducible values, which were checked manually using Fiji (ImageJ v2·0, open-source software).^1^ Next, the detected pixels in both channels are compared, and only the matching ones are used to generate the output image and metrics. The average amount of detected BC particles in breast milk was normalized for the imaging volume using the focal volume estimated from the spatial resolution of the optical system (810 nm, identical settings, 20x/0.8): w_x_ = w_y_ = 0.48 μm and w_z_ = 2.37 μm, defined as, respectively, the sizes of the point spread function in the XY-plane and along the optical axis (z-axis) (radius at the 1/e^2^ intensity level). Finally, the total relative number, i.e., the number of detected BC particles per mL breast milk, was defined.

### Lipid measurements

About 2 mL of breast milk was weighed and liquid–liquid extracted with 5 mL (two times) of n-hexane:acetone (3:1, vol/vol). After extraction, 1 mL of extract was transferred to a small precleaned metal tray and dried at 110° C for 1 h. The weight difference of the tray was further used for lipid content calculation (in %).

### Ambient exposure measurements

The ambient PM_2.5_, PM_10_, and NO_2_ concentrations (in μg/m^3^) were determined with a spatial interpolation model based on the maternal residential address. Full details on the model are described elsewhere (24). Briefly, land cover data from the CORINE land cover dataset were used to interpolate pollution data from the official fixed monitoring stations in Belgium, including 79, 80, and 93 stations for PM_2.5_, PM_10_, and NO_2_, respectively. This model provides interpolated air pollution values on a 4 × 4 km^2^ grid. We determined different exposure windows by computing the mean daily concentrations 1 week (i.e., recent exposure) and 1 month (i.e., medium-term exposure) before sampling. Residential distance (in meters) to a major road (i.e., a road with more than 10,000 motor vehicles per day) was calculated using geographic information system functions (ArcGIS 9.3; Esri Belux S.A., Wemmel, Belgium) based on the maternal residential address.

### Statistical analysis

The data was analyzed and visualized using R (version 4.3.0), RStudio software (version 2023.03.0), and GraphPad Prism (version 9). All data are represented as means ± standard deviation (SD). To assess the relation between residential exposures or breast milk fat content and BC load in human breast milk, Spearman’s correlation coefficients were determined. The normal distribution was examined using the Shapiro–Wilk test.

## Results

### BC load in human breast milk

**Figure 1:**
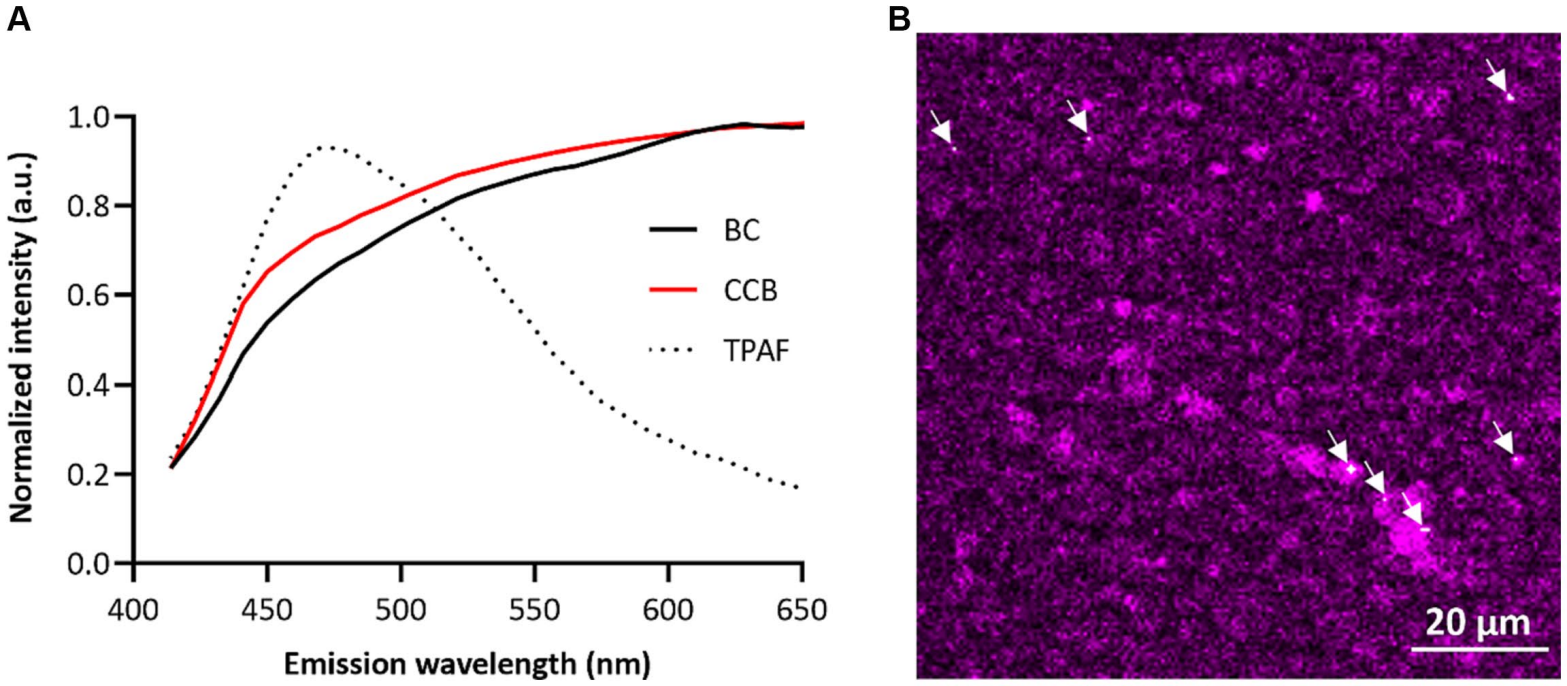
Evidence of BC particles in human breast milk. **(A)** Emission fingerprinting in human breast milk of BC particles, reference particles (CCB), and two-photon autofluorescence (TRAF) under femtosecond-pulsed illumination. **(B)** Localization of BC particles in human breast milk. Autofluorescence of human breast milk is given in magenta, and BC particles are shown in white (indicated by the white arrowhead). Scale bar: 20 μm.

Human breast milk samples were obtained from mothers who were on average 32.1 ± 3.9 years old and most of them had a BMI between 18 and 25 kg/m^2^ (75.0%). To confirm the carbonaceous nature of the particles, BC fingerprinting was performed. The emission fingerprint of identified BC particles in human breast milk resembled the signal of the reference particles (i.e., CCB) (Figure 1A). BC particles were found in all studied samples (Figure 1B), with an average of 8.8 × 10^5^ ± 4.3 × 10^5^ particles per mL of human breast milk, ranging from 3.4 × 10^5^ to 1.6 × 10^6^. BC load and the fat content of the human breast milk samples were not significantly correlated (*r* = 0.38; *p* = 0.36).

### BC load in human breast milk and residential exposures

**Figure 2:**
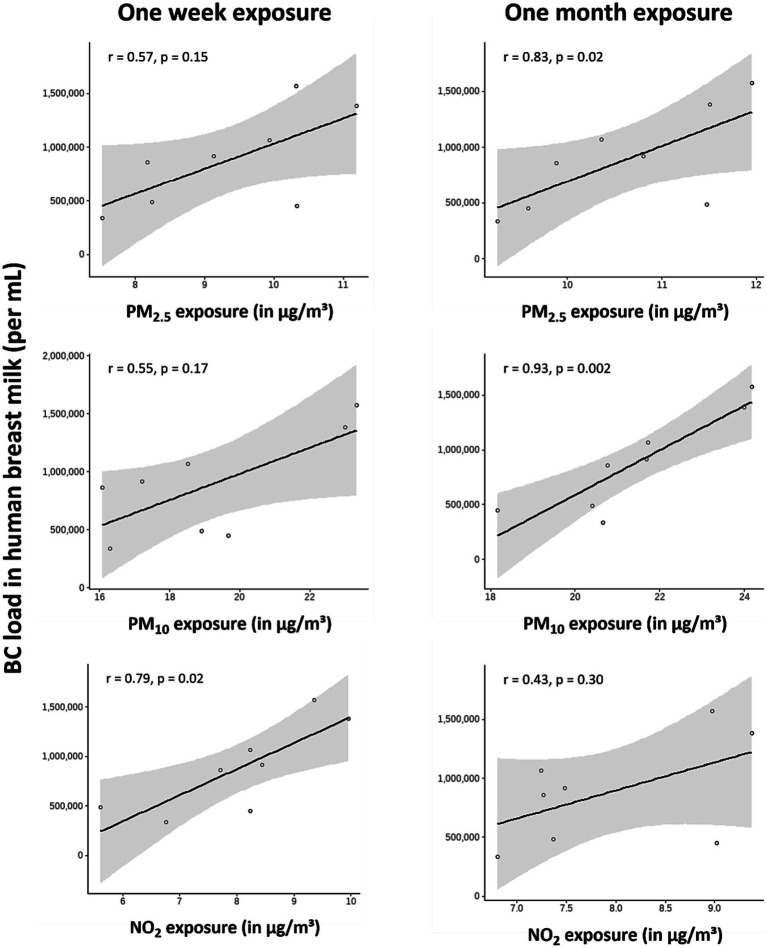
Correlation between BC load in human breast milk and maternal residential air pollution exposures 1 week before sampling (i.e., recent) or 1 month before sampling (i.e., medium-term). Spearman’s correlation coefficients were obtained for all exposures. The solid lines indicate the regression lines with the 95% CIs (grey areas).

The average maternal residential recent and medium-term air pollution exposures are provided in Table 1, while sample-specific information on residential exposures is listed in Supplementary Table S1. BC loads in human breast milk were strongly and positively correlated with recent maternal residential NO_2_ exposure (*r* = 0.79; *p* = 0.02). Furthermore, medium-term maternal residential PM_2.5_ (*r* = 0.83; *p* = 0.02) and PM_10_ (*r* = 0.93; *p* = 0.0002) exposure were very strongly correlated with BC load in human breast milk (Figure 2). Residential proximity to a major road, ranging from 55.3 to 1775.9 meters (Supplementary Table S1), was not significantly associated with BC load in human breast milk (*r* = −0.62; *p* = 0.11; Supplementary Figure S1).

**Table 1:** Average maternal residential air pollution exposures (in μg/m^3^).

	Recent^*^ exposure (μg/m^3^)			Medium-term^$^ exposure (μg/m^3^)		
	Mean ± SD	Min	Max	Mean ± SD	Min	Max
PM_2.5_	9.4 ± 1.3	7.5	11.2	10.6 ± 1.0	9.3	12.0
PM_10_	19.1 ± 2.8	16.1	23.3	21.5 ± 2.0	18.2	24.2
NO_2_	8.0 ± 1.4	5.6	10.0	7.9 ± 1.0	6.8	9.4

^*^Recent: 1 week before sampling; ^$^medium-term: 1 month before sampling.

## Discussion

Our pilot study showed, for the first time, that BC is present in human breast milk and found a strong association with ambient air pollution levels, suggesting that BC particles may be transferred to breast milk after inhalation through the mother. Here, we propose a novel pathway through which BC particles can directly enter the infant’s system, in addition to being exposed via inhalation.

The Developmental Origins of Health and Disease (DOHaD) hypothesis states that environmental exposures during crucial stages of development and growth may have significant consequences on an individual’s short- and long-term health (25). Therefore, it is important to understand the optimal environmental factors, such as diet, during infancy. As optimized nutrition in the first 1,000 days of life (i.e., from conception through the second birthday) is critical for the healthy development of the newborn (26), understanding to which extent environmental pollutants reach the infant through breastfeeding is critical for identifying strategies to minimize exposure and protect mothers’ and infants’ health and well-being. Mothers are exposed to various environmental pollutants on a daily basis, potentially contaminating human breast milk and affecting children’s early development (27). Most of the published studies focused on the presence of environmental chemicals, such as polychlorinated biphenyls, persistent organic pollutants, PFAS, organochlorine pesticides, polybrominated diphenyl esters, and phthalates (8, 13). However, human studies investigating the presence of environmental particles are scarce. Ragusa and colleagues detected microplastics for the first time in human breast milk, with a size ranging from 3 to 12 μm (28). Furthermore, nanoplastics had a high binding affinity to secretory immunoglobulin A, an antibody in human breast milk that plays a crucial role in disease protection, potentially interfering with the development of the infant’s immune system (29).

While studies investigating environmental particles in human breast milk are limited, several rodent studies have shown that nanoparticles could be transferred to the offspring through breast milk (20–22, 30, 31). In rats, silver nanoparticles were distributed to breast milk (20, 31) and subsequently to the brains of breastfed pups (20). In addition, zinc oxide nanoparticles were distributed to the intestine and liver of rat pups via breast milk (22). Furthermore, quantum dots (i.e., semiconductor nanocrystals) were detected in the breast milk of rats and the stomach and intestine of their offspring, inducing growth restriction (30). In mice, metal oxide nanoparticles were found in breast milk and induced growth retardation in their offspring (21). To date, no research has focused on the presence of traffic-related particulate matter, such as BC, in human breast milk, making our study the first to provide evidence that BC can be detected in this human biological matrix.

A study assessing the deposition and retention of ultrafine carbon particles in the respiratory tract found that 75% of inhaled carbon particles in healthy nonsmokers were persistent in the airways 1 day after exposure. However, only about 3% of the carbon particles in the lung periphery were cleared within 24 h (32). Due to the stability of the carbon particles, long-term retention in the maternal lung periphery is expected. Although this might increase the inter-individual variability of BC load, we found a strong association between ambient air pollution exposures and BC load in human breast milk. We observed a stronger correlation between BC load in human breast milk and ambient PM_10_ levels than between BC load in human breast milk and ambient PM_2.5_ concentrations. Although typically more than 90% of BC resided in the PM_2.5_ size fraction, a one-year measurement period of BC, PM_2.5_, and PM_10_ in Finland showed that on an annual level, BC accounted for 14 ± 8% and 7 ± 4% of the total PM_2.5_ and PM_10_, respectively (33). Furthermore, the study by Gong W and colleagues (34) reported a similarly high correlation between BC and PM_2.5_ and PM_10_. We observed two strong correlations, yet given the restricted sample size, we are careful not to overinterpret this difference. In contrast with our study, living close to a major road was associated with higher urinary BC load (17). We found a similar trend in our results, but did not reach the significance level, possibly due to the limited sample size. Bongaerts et al. (19) reported the BC load in maternal blood to range between ~3.0 × 10^4^ and 1.6 × 10^6^ particles per mL blood, which is slightly lower compared with the BC load in human milk in our study, although our limited sample size should be considered in this comparison.

Notably, BC particles might have an affinity for attaching to the fat globules within human breast milk. Given that the fat content in human breast milk varies within feeds (i.e., foremilk and hindmilk), during lactation, and depends on the individual maternal characteristics (35, 36), this could lead to a higher variability in BC load in human breast milk samples. However, we did not find a significant correlation between the fat content and BC load in human breast milk.

While it may be concerning to find environmental pollutants in human breast milk, it is crucial to emphasize that this should not undermine breastfeeding. Numerous studies and reviews have consistently stated that the risks of not breastfeeding far outweigh the potential risks associated with the exposure to pollutants (37, 38) and also the data of the current study do not argue against breastfeeding. Nonetheless, some studies have hinted at the possibility that pollutants in human breast milk could somewhat diminish the developmental advantages of breastfeeding (9). However, it’s worth noting that this attenuation is not statistically significant when considering other influential factors in child development, such as parental influence and home environment, after appropriate control measures are applied (9). Thus, instead of discouraging breastfeeding, these findings should serve as a clarion call for governments and policymakers to take decisive action on environmental health. By addressing and mitigating the sources of pollution in our environment, we can create a safer and healthier world for children to grow up in. This endeavor aligns seamlessly with the United Nations’ Sustainable Development Goals, which aim to ensure that future generations have the best chance at healthy development within a sustainable and clean environment (39).

### Strengths and limitations

Our study has several strengths. We detected for the first time BC particles in human breast milk samples. Our biocompatible and label-free detection method enables the precise and highly sensitive detection of ambient BC particles in their biological context (23). We confirmed the carbonaceous nature of the BC particles in human breast milk through emission fingerprinting. In addition to PM_2.5_ and PM_10_ levels, we included ambient NO_2_ concentrations, as its concentration is above the average of the European concentration and is generally higher in the Flemish Region of Belgium, where road traffic is the leading source of NO_2_ emission, compared to the Walloon Region (40). Some limitations should also be acknowledged. In this pilot study, we used a limited sample size (*n* = 8) to assess the presence of ambient BC particles in human breast milk. A larger sample size is required to provide a better estimation of the variability of BC load in human milk. Nonetheless, our study showed that BC particles can reach the nursing infant through breastfeeding, as the particles were detected in all studied samples and were strongly correlated with modelled air pollution exposures. The study design does not allow to draw conclusions about the translocation of BC from the intestine to the blood. The potentially harmful effects of BC exposure can be caused by transfer to the circulation but can also be due to its ability to disrupt the milk or gut microbiome of the child. Although other sources of exposure (e.g., while commuting or at work) can influence an individual’s exposure to air pollutants, mother’s residential exposures were strongly and positively linked with particles present in maternal and cord blood (19), as well as in placental tissue (17–19), suggesting a suitable estimation of individual exposures.

## Conclusion

We found evidence of BC particles in human breast milk, suggesting a novel pathway through which BC particles can directly enter the infant’s system. While the presence of environmental BC particles in human breast milk is a cause for concern, it also serves as a call to action. The findings of our study could have important implications for public health policies aimed at promoting breastfeeding and protecting infants from the potential harms of environmental pollutants.

## Data availability statement

The data used in this study are not publicly available because they contain information that could compromise research participant privacy but are available within General Data Protection Regulation restrictions from the corresponding author upon reasonable request.

## Ethics statement

The studies involving humans were approved by the Ethical Committees of Hasselt University (Diepenbeek, Belgium) and the East-Limburg Hospital (Genk, Belgium). The studies were conducted in accordance with the local legislation and institutional requirements. The participants provided their written informed consent to participate in this study.

## Author contributions

CC: Conceptualization, Formal analysis, Investigation, Visualization, Writing – original draft, Writing – review & editing. EB: Investigation, Validation, Writing – review & editing. KV: Validation, Visualization, Writing – review & editing. BR: Data curation, Writing – review & editing. AS: Formal analysis, Writing – review & editing. ET: Writing – review & editing. GP: Investigation, Writing – review & editing. MA: Methodology, Writing – review & editing. TN: Conceptualization, Methodology, Writing – review & editing. MP: Conceptualization, Supervision, Writing – review & editing.

## Footnotes

^1^ http://fiji.sc/Fiji
