# Supplementary material for: Black carbon particles in human breast milk: assessing infant’s exposure
Source: Front Public Health. 2024 Jan 17;11:1333969. doi: 10.3389/fpubh.2023.1333969 (PMC10828029; doi:10.3389/fpubh.2023.1333969)
Supplement: Supplementary file 1 [file Data_Sheet_1.docx]

**SUPPLEMENTAL INFORMATION**

SUPPLEMENTARY TABLES

**Supplementary Table S1: Black carbon load in human breast milk with matching residential exposures.** ^*^Recent: one week before sampling; ^$^medium-term: one month before sampling.

| **ID** | **BC load (particles/mL)** | **Recent^*^ exposure (µg/m³)** | | | **Medium-term^$^ exposure (µg/m³)** | | | **Distance to a major road (m)** |
| --- | --- | --- | --- | --- | --- | --- | --- | --- |
|  |  | **PM_2.5_** | **PM_10_** | **NO_2_** | **PM_2.5_** | **PM_10_** | **NO_2_** |  |
| 1 | 4.5 x 10^5^  ± 2.3 x 10^5^ | 10.3 | 19.7 | 8.2 | 9.6 | 18.2 | 9.0 | 988.2 |
| 2 | 1.6 x 10^6^  ± 5.0 x 10^5^ | 10.3 | 23.3 | 9.4 | 11.9 | 24.2 | 9.0 | 295.5 |
| 3 | 1.4 x 10^6^  ± 2.7 x 10^5^ | 11.2 | 23.0 | 10.0 | 11.5 | 24.0 | 9.4 | 357.2 |
| 4 | 1.1 x 10^6^  ± 5.7 x 10^5^ | 9.9 | 18.5 | 8.2 | 10.4 | 21.7 | 7.2 | 105.3 |
| 5 | 4.9 x 10^5^  ± 2.6 x 10^5^ | 8.2 | 18.9 | 5.6 | 11.5 | 20.4 | 7.4 | 334.5 |
| 6 | 3.4 x 10^5^  ± 3.5 x 10^5^ | 7.5 | 16.3 | 6.8 | 9.3 | 20.7 | 6.8 | 1775.9 |
| 7 | 9.2 x 10^5^  ± 7.2 x 10^5^ | 9.1 | 17.2 | 8.4 | 10.8 | 21.7 | 7.5 | 55.3 |
| 8 | 8.6 x 10^5^  ± 5.2 x 10^5^ | 8.2 | 16.1 | 7.7 | 9.9 | 20.8 | 7.3 | 322.8 |

SUPPLEMENTARY FIGURES


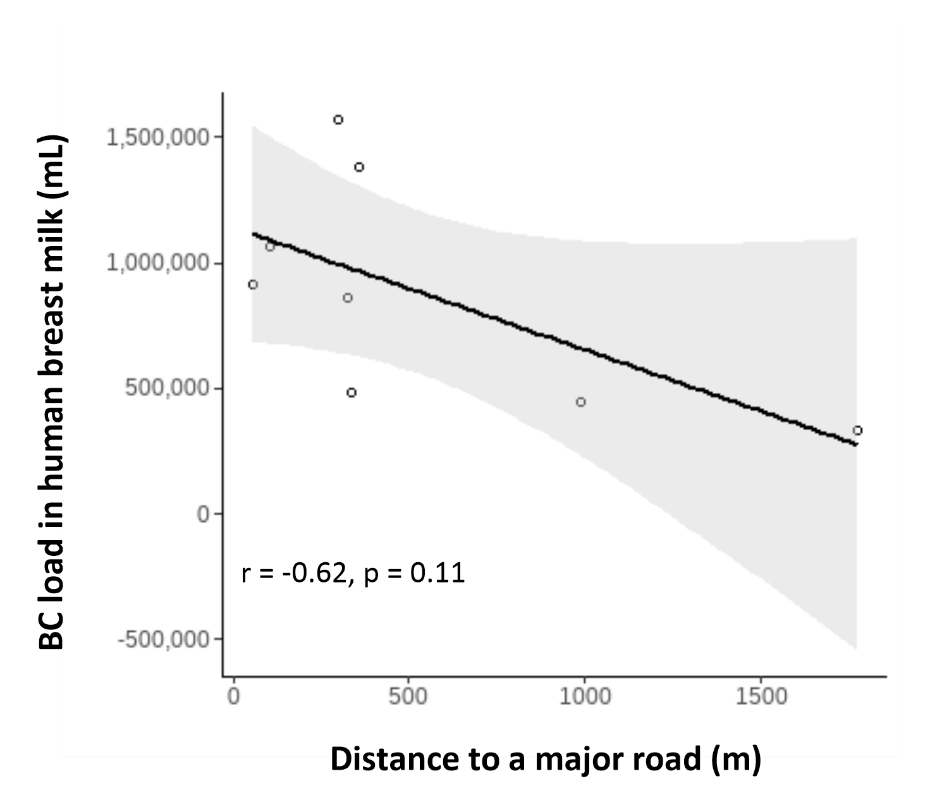


**Supplementary Figure S1: Spearman correlation between BC load in human breast milk and residential distance to a major road (m).** The solid line indicates the regression line with the 95% CI (grey area).
